# Hydroxypropyl Methylcellulose E15: A Hydrophilic Polymer for Fabrication of Orodispersible Film Using Syringe Extrusion 3D Printer

**DOI:** 10.3390/polym12112666

**Published:** 2020-11-12

**Authors:** Pattaraporn Panraksa, Suruk Udomsom, Pornchai Rachtanapun, Chuda Chittasupho, Warintorn Ruksiriwanich, Pensak Jantrawut

**Affiliations:** 1 Department of Pharmaceutical Sciences, Faculty of Pharmacy, Chiang Mai University, Chiang Mai 50200, Thailand; pattaraporn.prs@gmail.com (P.P.); chuda.c@cmu.ac.th (C.C.); Yammy109@gmail.com (W.R.); 2 Biomedical Engineering Institute, Chiang Mai University, Chiang Mai 50200, Thailand; suruk_u@cmu.ac.th; 3 Division of Packaging Technology, School of Agro-Industry, Faculty of Agro-Industry, Chiang Mai University, Chiang Mai 50100, Thailand; pornchai.r@cmu.ac.th; 4 Cluster of Research and Development of Pharmaceutical and Natural Products Innovation for Human or Animal, Chiang Mai University, Chiang Mai 50200, Thailand

**Keywords:** 3D printing, syringe extrusion 3D printing, hydroxypropyl methylcellulose, orodispersible film, phenytoin

## Abstract

Extrusion-based 3D printing technology is a relatively new technique that has a potential for fabricating pharmaceutical products in various dosage forms. It offers many advantages over conventional manufacturing methods, including more accurate drug dosing, which is especially important for the drugs that require exact tailoring (e.g., narrow therapeutic index drugs). In this work, we have successfully fabricated phenytoin-loaded orodispersible films (ODFs) through a syringe extrusion 3D printing technique. Two different grades of hydroxypropyl methylcellulose (HPMC E5 and HPMC E15) were used as the film-forming polymers, and glycerin and propylene glycol were used as plasticizers. The 3D-printed ODFs were physicochemically characterized and evaluated for their mechanical properties and in vitro disintegration time. Then, the optimum printed ODFs showing good mechanical properties and the fastest disintegration time were selected to evaluate their drug content and dissolution profiles. The results showed that phenytoin-loaded E15 ODFs demonstrated superior properties when compared to E5 films. It demonstrated a fast disintegration time in less than 5 s and rapidly dissolved and reached up to 80% of drug release within 10 min. In addition, it also exhibited drug content uniformity within United States Pharmacopeia (USP) acceptable range and exhibited good mechanical properties and flexibility with low puncture strength, low Young’s modulus and high elongation, which allows ease of handling and application. Furthermore, the HPMC E15 printing dispersions with suitable concentrations at 10% *w/v* exhibited a non-Newtonian (shear-thinning) pseudoplastic behavior along with good extrudability characteristics through the extrusion nozzle. Thus, HPMC E15 can be applied as a 3D printing polymer for a syringe extrusion 3D printer.

## 1. Introduction

Over the last few decades, there has been a growing interest in the use of three-dimensional (3D) printing technology within the medical and pharmaceutical fields to fabricate the customizable solid dosage forms that suit different needs, preferences and individual characteristics of each patient [1]. Three-dimensional printing is a manufacturing method that can fabricate 3D-printed products of any shape and size on-demand from digital design software through depositing materials layer-by-layer [2]. This technology involves three commonly used techniques: printing-based inkjet (IJ) systems, nozzle-based deposition systems or extrusion (solid or semi-solid)-based printing technique and laser-based writing systems. Among these, the extrusion-based printing technique has been recognized as the most popular technique for the fabrication of solid oral dosage forms owing to their excellent capability to print with a wider selection of polymers and drugs at room temperature and the capability to incorporate high amounts of drugs with low-cost [1,3]. Numerous studies have been performed regarding the benefits of extrusion-based 3D printing to design various novel dosage forms such as polypills, gastro-floating tablets and orodispersible films (ODFs) [4]. ODFs are the relatively novel dosage form prepared by using hydrophilic polymers and designed to rapidly disintegrate within a minute in the buccal cavity, without requiring water [5]. This dosage form exhibits several advantages over other oral dosage forms, including ease of administration to pediatric and geriatric patients experiencing dysphagia (swallowing difficulty), dose flexibility and improving the bioavailability of drugs due to high vascularity and high permeability in the buccal cavity [6]. The major advantages of preparing an ODF by 3D printing over the standard film solvent casting are the ability to print objects with different filling (hollow, matrix or full) and the dose of drugs can be controlled by calculating the material consumption during the resizing of the printed object at the design stage, which is proper for personalized therapy. Moreover, 3D-printed films can be formulated with less amount of time.

A variety of hydrophilic polymers such as polyvinyl alcohol (PVA), polyvinylpyrrolidone (PVP), polyethylene glycol (PEG), hydroxypropyl cellulose (HPC) and hydroxypropyl methylcellulose (HPMC) are used as film-forming polymers for the preparation of ODFs, and most of them can also be used as printing materials for extrusion-based 3D printers [7,8]. Hydroxypropyl methylcellulose (HPMC), also known as hypromellose, is widely implemented in pharmaceutical manufacturing as a binder, thickening agent, hydrophilic matrix material and film-forming material. It is classified into several grades based on viscosity, degree of hydroxypropyl substitution and degree of methoxy substitution. The low viscosity HPMC grades (e.g., HPMC E3, HPMC E5 and HPMC E15) are often used for ODFs preparation and suitable for extrusion-based 3D printing of oral dosage forms [8,9,10]. Moreover, the use of HPMC, which is a hydrophilic polymer, can further be advantageous in terms of enhancing the solubility and dissolution of poorly water-soluble drugs in the manufacture of solid dispersion. However, there are still limited studies available on the preparation of 3D-printed ODFs while using low viscosity HPMC as a film-forming polymer. In a previous study, levocetirizine dihydrochloride ODFs consisting of HPMC E15 and pregelatinized starch were prepared using semi-solid extrusion (SSE) 3D printer. The 3D-printed ODFs exhibited good flexibility and rapid drug release in vitro by dissolving completely in two minutes [11]. In addition, previous work on the extrusion-based 3D-printing of HPMC in the pharmaceutical field can be found [12]. The extrusion-based (fused-deposition modeling) 3D printer was used to fabricate 3D-printed tablets by using their developed HPMC filament.

Phenytoin, which was selected as a model drug in this study, is an antiepileptic drug widely used in the treatment of partial seizures, generalized seizures and status epilepticus [13]. It belongs to the Biopharmaceutical Classification System (BCS) class II drug in which its bioavailability is limited due to poor water solubility (32 μg/mL). Various approaches were employed to overcome the solubility problem. Solid dispersion of the drug in a hydrophilic polymer is also one of the promising techniques for enhancing its solubility. There are many available dosage forms of phenytoin in the market, such as oral suspension, chewable tablets, capsules, and intravenous injections. However, the commercial production of orodispersible phenytoin dosage form has not yet been available. The development of phenytoin ODF has numerous advantages over conventional dosage forms such as convenience for patient administration, accurate drug dosing, rapid onset of action with increased bioavailability due to bypassing hepatic first-pass effect and noninvasiveness. Moreover, phenytoin ODF can be used for dysphasic and schizophrenic patients and can be taken without water due to their ability to disintegrate within a few minutes to release medication in the mouth.

Consequently, the present study aimed to assess the possibility of phenytoin ODFs fabrication by syringe extrusion 3D printer using two different low viscosity grades of HPMC (HPMC E5 and HPMC E15) as film-forming polymers. The extrudability and printability of different grades of HPMC were investigated and discussed. Then, the developed 3D-printed ODFs were evaluated for their physicochemical properties, mechanical properties, in vitro disintegration time and in vitro release profiles. The most important impact of our work is the use of the syringe extrusion 3D printer developed by Biomedical Engineering Institute, Chiang Mai University, Thailand and the use of a polymer (HPMC E15) with optimum viscosity as a single printing material to fabricate phenytoin-loaded ODFs for leading to personalized medicine. Until now, there has been no previous study/experiment that has used this syringe extrusion 3D printer to fabricate 3D-printed products. Our syringe extrusion 3D printer was developed to print varieties of fluid gels like materials, such as hydrogels and pastes. Moreover, it can control the printing material temperature by using a temperature control system on the syringe socket. This temperature control system will help control the viscosity of printing material and keep the printing material in a semi-solid state, which makes the material printable through the 3D printer.

## 2. Materials and Methods

### 2.1. Materials

The model drug, 5,5-diphenylhydantoin (phenytoin: PT), with purity >98%, was purchased from Merck (Darmstadt, Germany). Hydroxypropyl methylcellulose E5 (HPMC E5, AnyCoat^®^-C AN5, substitution type 2910, viscosity 5 mPa∙s) and Hydroxypropyl methylcellulose E15 (HPMC E15, AnyCoat^®^-C AN15, substitution type 2910, viscosity 15 mPa∙s) were purchased from Lotte Fine Chemical Co., Ltd. (Seoul, South Korea). Refined glycerin was purchased from Srichand United Dispensary Co., Ltd. (Bangkok, Thailand). Propylene glycol (PG) was purchased from Dow Chemical Company (Midland, MI, USA). Ethanol ≥95% (Bangkok Alcohol Industrial Co., Ltd., Bangkok, Thailand) and distilled water were used as the solvents for preparing the printing dispersions. All of the other reagents were analytical grade.

### 2.2. Determination of Extrudability and Printability

#### 2.2.1. Rheological Characterization

To investigate the printability of the different concentrations of two different grades of HPMC that were proposed for use in syringe extrusion 3D printing, the rheological and dimensional accuracy tests of 10, 12.5, 15 and 20 % *w/v* of HPMC E5 and HPMC E15 were conducted. The samples were prepared by dispersing HPMC E5 and HPMC E15 in ethanol–water mixtures (9:1) and stirred for 4 h with a magnetic stirrer.

The rheological behaviors of HPMC E5 and HPMC E15 were investigated using a plate-and-plate Brookfield Rheometer (Brookfield Rheometer R/S, P25 DIN plate, Brookfield engineering laboratories, Middleboro, MA, USA). The measurements were carried out by measuring the shear stress and viscosity with varying the shear rate ranging from 0 to 100 s⁻^1^ at 25 °C. The gap between plate and base was set at 1 mm. All the experiments were carried out in triplicate. The flow behavior index (n) and consistency coefficient (K) were calculated using the power-law model equation:

$$\tau =K{\dot{\gamma }}^{n}$$

where τ is the shear stress (Pa), $\dot{\gamma }$ is the shear rate (s^−1^), and K is a consistency coefficient (Pa∙s*^n^*)

#### 2.2.2. 3D Printer, Design and Printing Parameters

**Figure 1:**
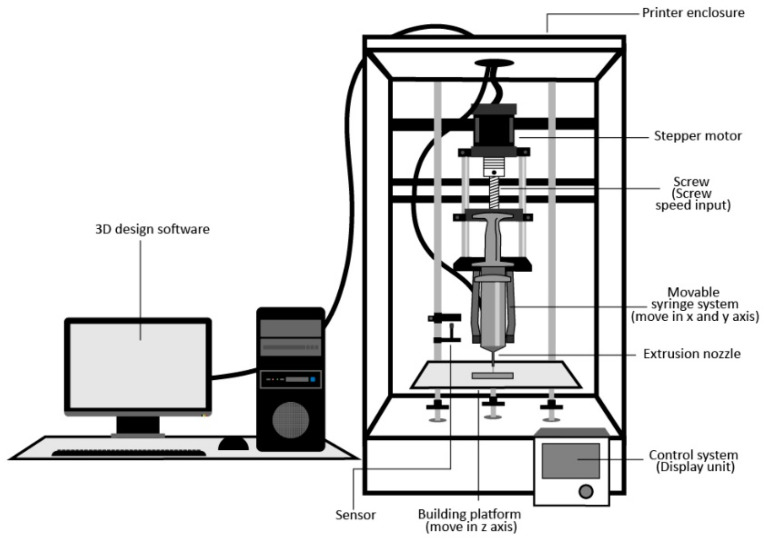
Schematic diagram of syringe extrusion 3D printer.

In this study, the syringe extrusion 3D printer (Figure 1) developed by the Biomedical Engineering Institute of Chiang Mai University was used to fabricate the 3D-printed ODFs. This customized syringe extrusion 3D printer is based on a core-XY 3D printer in which an extrusion nozzle can move in an X and Y axis and a separate building plate on a *Z*-axis while printing layer-by-layer for generating 3D structure to prevent vibration on the sample. A custom syringe-based extruder was designed and built in-house for precision deposition. In Figure 1, the syringe-based extruder used a stepper motor to move a plunger of a 10 mL syringe via a direct lead screw drive. The syringe extrusion 3D printer was controlled by a computer and a user interface on the printer. Before 3D printing, an object was designed using an open-source program and was divided into numerous two-dimensional (2D) layers with a defined thickness, infill and speed of printing. These 2D layers can be piled up by selectively adding the desired materials in a highly reproductive layer-by-layer manner under the instruction of computer-aided design (CAD) models.

**Figure 2:**
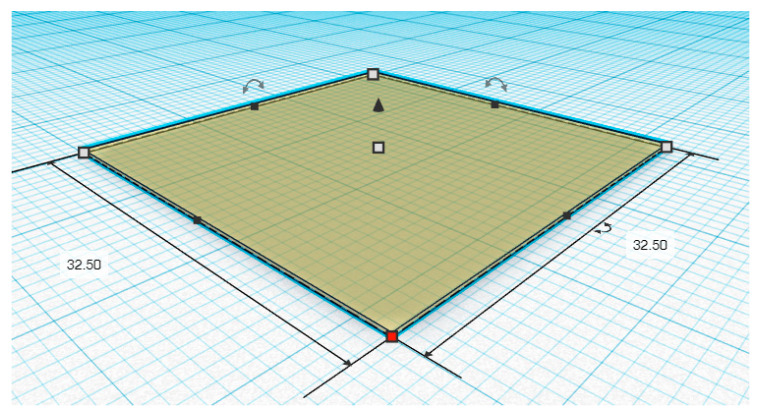
Computer-aided design (CAD) of the 3D-printed orodispersible films (ODF) (length 32.50 mm, width 32.50 mm and height 0.19 mm).

The 3D printing process was performed using a syringe extrusion 3D printer. Initially, as shown in Figure 2, the 3D designs and models of the printed ODF with the dimension of 32.5 mm width × 32.5 mm length × 0.19 mm height were obtained using Tinkercad^®^ software (2020, Autodesk Inc., San Rafael, CA, USA). The 3D models were constructed by a simplified constructive solid geometry method and exported as a 3D printer readable stereolithography (.stl) file format to Repetier-Host software version 2.1.6 (Hot-World GmbH & Co. KG., Willich, Germany). Then, the .stl file was sliced and converted to a 3D printable code (G-code) by the open-source Slic3r software version 1.3.1 (GNU Affero general public license, version 3). Thereafter, the samples were transferred into a 10 mL disposable syringe (14.5 mm diameter), and the 3D models were printed with a syringe extrusion 3D printer equipped with a single head printing extruder nozzle with a diameter of 0.51 mm (21 G). The printing process was conducted at 25 °C, 10 mm/s printing speed and 120 mm/s nozzle traveling speed, and the printing parameters were preset as the following: the layer height was 0.19 mm, the fill angle was 45°, the perimeters were 2, and the infill was defined as rectilinear with 100% ratio (For the measurement of the diameter of printed filaments, the infill was set as 0% of the volume, and the perimeter was 1).

#### 2.2.3. Dimensional Accuracy Tests

The diameters of printed filaments (0% infill) and area of printed ODFs (100% infill) were determined using photos taken by a digital camera (Canon EOS 750D with an 18–55 mm lens, Canon, Inc., Tokyo, Japan) and measured with ImageJ software version 1.52 (National Institutes of Health, Bethesda, MD, USA; available at https://imagej.nih.gov/ij/index.html). Then, the shape fidelity factor (SFF), which is the ratio between the 3D-printed area and CAD model area, was calculated using the following equation:

$$Shape\;fidelity\;factor=\;\frac{Printed\;area\;\left(cm^{2}\right)}{CAD\;model\;area\;\left(cm^{2}\right)}$$

### 2.3. Fabrication of 3D-Printed Orodispersible Film

In order to prepare the printing dispersions for fabrication, the following steps were taken. First, HPMC and phenytoin were dispersed in specific proportions as listed in Table 1 in the mixed solvent of ethanol and distilled water (9:1, *v/v*) and stirred for at least 3 h with a magnetic stirrer at room temperature (25 ± 2 °C). Subsequently, the two different plasticizers (glycerin and propylene glycol) were added into the dispersions at 10% *w/w* of the polymer. The dispersions were gently stirred for 10 min and then left until all of the air bubbles disappeared. Ten milliliters of the dispersion were loaded into a 10 mL disposable syringe attached with 21 G needle tips and then printed with the parameters described in Section 2.2.2. The total printing time was approximately 3.5 min for each film. After printing, the printed ODFs were placed at room temperature for 15–30 min to complete the drying process.

### 2.4. Characterization of 3D-Printed ODFs

#### 2.4.1. Morphological Characterization

The morphology of the printed ODFs was investigated by scanning electron microscopy (SEM) with a JEOL scanning electron microscope (JSM-5410LV, JEOL, Ltd., Peabody, MA, USA) at 10 kV under low vacuum mode. The film characterizations were performed without any coating solution at magnifications of ×500. The thickness and surface of the films were evaluated.

#### 2.4.2. Film Thickness and Weight Variation

The thickness and weight variation were determined by selecting ten printed ODFs randomly. The thickness of each 3.25 cm × 3.25 cm sized film was measured at three points by an outside micrometer (3203-25A, Insize Co, Ltd., Suzhou New District, Jiangsu, China). Weight variation was evaluated by an analytical balance (PA214, Ohaus Corporation, Parsippany, NJ, USA). The average thickness (in mm) and weight (in g) of printed ODFs with the standard deviation were calculated. The measurements were conducted in triplicate.

#### 2.4.3. Mechanical Properties of 3D-Printed ODFs

**Figure 3:**
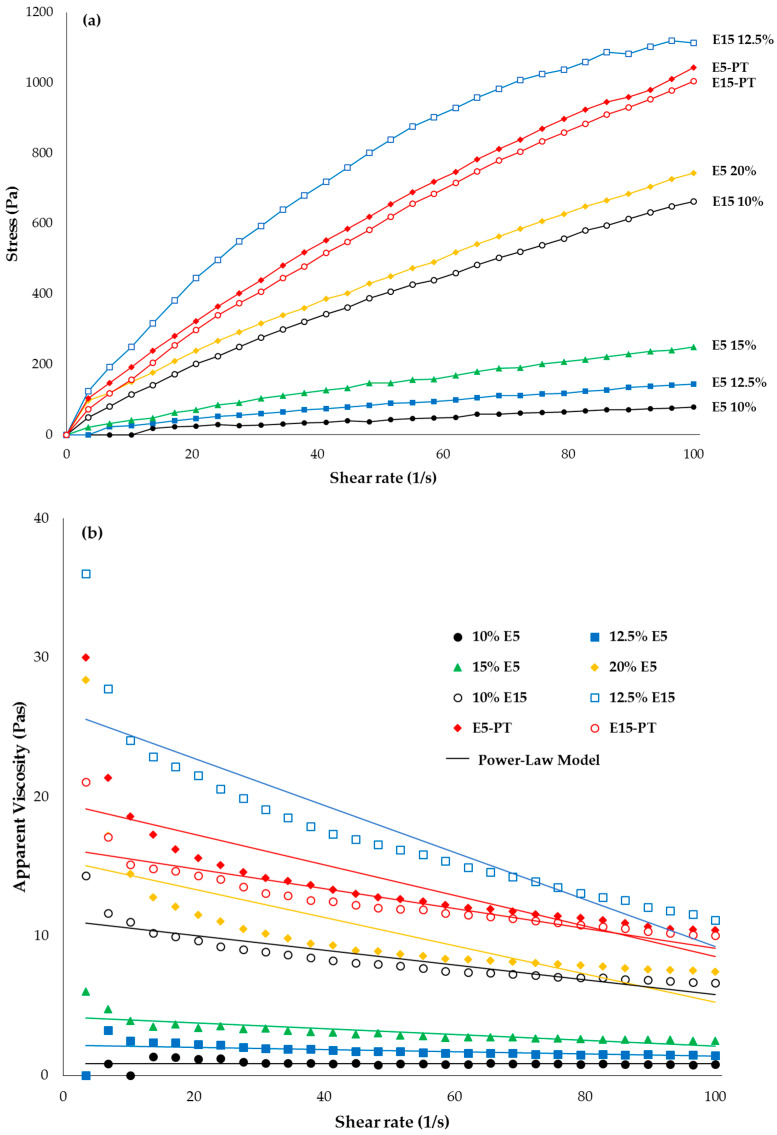
Rheological behaviors of hydroxypropyl methylcellulose (HPMC) E5 and HPMC E15 at different concentrations: (**a**) stress versus shear rate; (**b**) viscosity versus shear rate and fitting power-law model.

Mechanical strength tests were carried out on square ODFs pieces (3.25 cm × 3.25 cm) with a texture analyzer TX.TA plus (Stable Micro Systems, Surrey, UK) using a cylindrical stainless probe (2 mm diameter) with a plane flat-faced surface (probe contact area =3.14 mm^2^). The film was fixed on the plate with a cylindrical hole of 9.0 mm-diameter (area of the sample holder hole =63.56 mm^2^), and a cylindrical probe was moved down at a velocity of 1.0 mm/s (Figure 3). Measurement started when the probe had contacted the sample surface (triggering force). The probe moved on at constant speed until the film was torn. The maximum force (N), distance (mm) and slope of the linear region of the force–time curve (N/s) were recorded. All of the experiments were conducted in triplicate for each sample at room condition (25 °C, 70% relative humidity). The mechanical strength of the film was characterized by puncture strength (MPa), elongation to break (%) and Young’s modulus (MPa), which were calculated using the following equations [14,15]:

$$Puncture\;strength=\;\frac{F_{max}}{Ar_{p}}$$

where $F_{max}$ is the maximum force required to tear the film (N) and $Ar_{p}$ is the probe contact area (mm^2^).

$$Elongation\;to\;break\;=\;\left(\frac{\sqrt{{a^{\prime }}^{2}+b^{2}}+r}{a}-1\right)\;\times \;100$$

where a is the radius of the film in the radius of sample holder cylindrical hole, $a^{\prime }$ is the length of the film sample is not torn by the probe (a−r), b is the probe displacement, and r is the probe radius.

$$Young^{\prime }s\;modulus=\;\frac{Slope\;of\;force-time\;curve\;\left(N/s\right)}{Film\;thickness\;\times Probe\;speed}$$

#### 2.4.4. In Vitro Disintegration Time Study

The disintegration test method used in this study was modified from Preis et al. (2014) [16]. The printed ODF was clamped between the sample holder and the magnetic clip (attached to the bottom side of the film). The magnetic clip had a weight of 3 g (0.03 N) that represented the approximate minimal force applied by the human tongue. Then, the attached film was half-immersed (50%) in 70 mL of simulated salivary fluid pH 6.8, which was prepared according to Marques et al. (2011) [17], at 37 ± 0.5 °C. The time required for the film to break and the magnetic clip to drop down was recorded visually and noted as in vitro disintegration time. All studies were performed in triplicate for each formulation. The optimum formulations in terms of mechanical properties and disintegration time were selected for further experiments.

### 2.5. Phenytoin Content

Three printed ODFs of size 3.25 × 3.25 cm dimension of each selected formulation (E5-PT-G and E15-PT-G) were taken in the vials containing 5 mL of distilled water and stirred at 600 rpm until the film was completely dissolved, after which 15 mL of ethanol was added and then set aside to ensure complete solubility of phenytoin. The solution was properly diluted with Tris buffer pH 6.8 with 1% *w/v* sodium lauryl sulfate (SLS) [18] and filtered through a 0.45 µm membrane filter. The phenytoin content was then determined by using HPLC. An HPLC system (HP 1100 Series HPLC, Agilent Technologies, Inc., Santa Clara, CA, USA) equipped with a Capcell Pak AQ 250 mm × 4.6 mm C18 column having a particle size of 5 µm (Shiseido Co. Ltd., Tokyo, Japan) was utilized. The mobile phase consisted of 23% *v/v* acetonitrile, 27% *v/v* methanol and 50% *v/v* of pH 3.0 phosphate buffer solution. The pump flow rate was maintained at 1.0 mL/min, and the injection volume was 10 µL. The UV detection wavelength was set as 240 nm. The phenytoin contents were calculated from the standard calibration curve of phenytoin in Tris buffer pH 6.8 with 1% *w/v* SLS, which demonstrated linearity with a high correlation coefficient (r^2^ = 0.9999). The following regression equation was obtained: *y =* 5.7615*x* − 1.8235, where *y* was the peak area and *x* was the concentration of phenytoin (µg/mL). All the measurements were performed in triplicate, and the average percentages of phenytoin content with the standard deviation were calculated.

### 2.6. In Vitro Phenytoin Release Study

The in vitro release test of printed ODFs was carried out in 300 mL of Tris with 1% *w/v* SLS buffer solution (pH 6.8) maintained at 37 ± 0.5 °C at a stirring rate of 50 rpm. At predetermined time intervals (1, 3, 5, 10, 15, 30 and 60 min), the samples were withdrawn, and then an equivalent volume of fresh dissolution medium was replaced in order to maintain sink conditions throughout the experiment. Withdrawn samples were filtered and analyzed by HPLC, as described in the drug content studies. The cumulative percentage of drug release was calculated using the standard calibration curve of phenytoin in Tris buffer pH 6.8 with 1% *w/v* SLS. All experiments were performed in triplicate.

### 2.7. Statistical Analysis

All the data were presented as mean ± SD. One-way ANOVA was used to evaluate the significance of differences at the significance level of *p*-value < 0.05. Statistical analysis was performed using SPSS software version 16.0 (SPSS, Inc., Chicago, IL, USA).

## 3. Results and Discussion

### 3.1. Rheological Characterization and Printability Study

The flowability and extrudability of printing dispersions are the important factors to ensure that the ODFs containing the required amount of phenytoin are printed precisely. The rheological behaviors of 10, 12.5, 15 and 20 % *w/v* of HPMC E5 and 10%, 12.5% HPMC E15 are shown in Figure 3. As observed in the figure, all the HPMC dispersions exhibited a non-Newtonian (shear-thinning) pseudoplastic behavior since their viscosity values decreased as well as shear stress increased when the shear rate increased from 0 to 100 s^−1^. Furthermore, the power-law model was fitted to the obtained results (shear stress–shear rate flow curves), and the power-law model parameters (flow behavior index (n) and consistency coefficient) are shown in Table 2. The results showed that n values were less than 1.0 (0.66 to 0.78) for all HPMC E5 and HPMC E15 dispersions indicating the shear-thinning pseudo-plasticity nature, and the n values decreased with increasing of polymer concentration, indicating more intense shear-thinning. For the consistency coefficient, the results exhibited that the consistency coefficient values of HPMC E5 were lower than those of HPMC E15 with the same concentration, indicating that HPMC E5 was less pseudoplastic and less viscous than HPMC E15. The viscosities of 10, 12.5, 15 and 20 % *w/v* of HPMC E5 were 0.80 ± 0.04, 1.70 ± 0.03, 3.02 ± 0.18 and 9.84 ± 0.08 Pas and the viscosities of 10 and 12.5 % *w/v* of HPMC E15 were 8.10 ± 0.51 and 16.85 ± 0.76 Pas, respectively. As expected, the viscosities were found to be increased with increasing concentration of each HPMC. Moreover, a higher viscosity value was also observed when phenytoin was incorporated into the formulations. In the case of E5 20% and E15 10% formulations, after phenytoin was incorporated, the viscosities were found to be 13.39 ± 0.74 and 12.17 ± 0.47 Pas, respectively. Additionally, the results indicated that, for lower molecular weight polymer (HPMC E5), the higher polymer mass was required to achieve the same viscosity as that of the high molecular weight polymer (HPMC E15).

According to Table 2, it was found that the diameters of the printed filaments were decreased when the concentration of HPMC E5 and HPMC E15 increased. The increase in the viscosity values could lead to the resolution improvement of printed ODFs in which the ODFs could be printed more accurately and more precisely. Shape fidelity factors of all printed formulations were close to one, suggesting that a stable shape of gel filament was deposited in the desired place during extrusion. Nonetheless, the results found that the addition of the drug could affect the viscosities and printability of the printing dispersion. The E15(12.5%)-PT formulation could not be extruded through the extruder nozzle smoothly due to its high viscosity and fast drying of the printing dispersion at the tip of the nozzle, ultimately causing a blockage of the extruder nozzle, whereas the E5(20%)-PT and E15(10%)-PT formulations were well extruded through the nozzle. Their diameters of printed filaments were found to be mostly close to the actual extruder nozzle diameters (0.51 ± 0.02 and 0.52 ± 0.01, respectively), thereby making them more suitable for this customized syringe extrusion 3D printer.

Thus, the results indicated that 20 and 10 % *w/v* were the suitable final polymer concentrations in printing dispersions for HPMC E5 and HPMC E15, respectively. Then, the E5(20%)-PT and E15(10%)-PT formulations, which exhibited similar rheological behavior and viscosity (approximately 12–13 Pas) and enabled extrusion through nozzles, were subsequently selected for plasticizers-loading and evaluated for their mechanical properties and disintegration time.

### 3.2. Film Preparation and Characterization

The control E5 and E15 ODFs were slightly yellowish to colorless and transparent, whereas the phenytoin-loaded ODFs (E5-PT, E5-PT-PG, E5-PT-G, E15-PT, E15-PT-PG and E15-PT-G ODFs) were white in color and opaque due to phenytoin content. It was also observed that E5-PT-G, E15-PT, E15-PT-PG and E15-PT-G ODFs were smooth, uniform and flexible while the E5-PT and E5-PT-PG were more brittle and easily cracked during the drying process due to lack of plasticizers or an inadequate amount of plasticizer. Moreover, it was observed that the printed surface of E5-PT-G, E15-PT, E15-PT-PG and E15-PT-G ODFs showed the rectilinear pattern printed diagonally without the interruption and did not exhibit any noticeable difference upon visual inspection.

Additionally, the thickness and weight variation of all printed films were carried out in order to ensure the consistency of the printing process and printed ODFs. The average thickness of all printed ODFs varied in the range of 0.013–0.061 mm while the average thickness of phenytoin-loaded ODFs varied in the range of 0.056–0.061 mm, which falls within a typical range for fast dissolving oral films (0.05–0.15 mm) and would be adequate for handling to avoid any discomfort when applied on the buccal mucosa [19]. Therefore, as can be observed in Table 3, the average thickness and weight of printed ODFs was increased when the drug and/or plasticizers were incorporated into the film formulations; however, there were no significant differences (*p* > 0.05) observed between the thickness and weight of phenytoin-loaded E5 ODFs and phenytoin-loaded E15 ODFs.

### 3.3. Mechanical Properties of 3D-Printed ODFs

The mechanical properties of all printed ODFs are displayed in Table 4, whereas the mechanical properties of E5-PT and E5-PT-G ODFs were not performed since they cracked during the drying process. The mechanical properties of the ODFs were taken to ensure that the ODFs possess suitable mechanical properties for easy handling and ease of removal from the packaging during use. In this study, the puncture strength, elongation to break and Young’s modulus were used as parameters to characterize the mechanical properties of printed ODFs. The puncture strength is a measure of the toughness of film or the required force to apply on the film surface and puncture the film until breaking point [20]. The elongation to break is a parameter that reflects the ductility and stretchability of film (the ability of a film to be stretched without being torn). The Young’s modulus is a parameter that reflects the elasticity and flexibility of film [21].

Mechanically, the films in which phenytoin was incorporated (E5-PT-G, E15-PT, E15-PT-PG and E15-PT-G ODFs) were found to have significantly lower puncture strength, elongation to break and Young’s modulus than the control E5 and E15 ODFs (*p* < 0.05), indicating that the E5-PT-G, E15-PT, E15-PT-PG and E15-PT-G ODFs were becoming less ductile and more flexible. This may be due to the disruption of the polymeric matrix continuity when drug particles were incorporated. This result was consistent with the previous studies showing that the addition of the drug to the formulations significantly influenced the mechanical properties of the film by decreasing the tensile strength and increasing the film’s brittleness [22,23,24].

Furthermore, this study also showed that, without any plasticizers, the phenytoin-loaded E5 films (E5-PT) could not be formed, whereas the E5 films with a plasticizer (E5-PT-G) showed no significant difference in the puncture strength, elongations to break and Young’s modulus value (*p* > 0.05) when compared with the phenytoin-loaded E15 films (E15-PT, E15-PT-G and E15-PT-PG). Additionally, it was observed that the E15-PT-PG and E15-PT-G ODFs showed slightly lower puncture strength, lower Young’s modulus and higher elongation to break than the films without any plasticizers (E15-PT ODFs), but the difference was not significant (*p* > 0.05). Thus, the results indicated that the addition of glycerin at a concentration of 10% (*w/w* of polymer) as a plasticizer in the ODFs formulation had the effect on improving the mechanical properties of both E5 and E15 formulations, whereas it may require a higher concentration of propylene glycol for E5 formulation in order to avoid cracking of the films, to induce sufficient flexibility and to obtain the similar mechanical properties as E5-PT-G ODFs. Due to the hydrogen-bonding capabilities of plasticizers (e.g., glycerin and propylene glycol), the addition of suitable amounts of plasticizers can improve the flexibility of films by allowing the interaction of hydrogen bonding between polymer chain functional groups and the plasticizing agent, resulting in the reduction of polymer–polymer interaction [25].

### 3.4. Disintegration Time

The results of in vitro disintegration time of E5-PT-G, E15-PT, E15-PT-PG and E15-PT-G ODFs are presented in Table 4. For the film disintegration tests, there is still no official guidance available for determining the disintegration time of ODFs in the pharmacopeia. Nevertheless, according to the United States Pharmacopeia (USP) and CDER guidance regarding the orally disintegrating tablets (ODTs), disintegration time (which may be applied to ODFs), a time limit of 30 s or less for disintegration is specified [26]. In this study, the results showed that E5-PT-G, E15-PT, E15-PT-PG and E15-PT-G ODFs disintegrated within 6 s, which passes the time limit for disintegration. Moreover, all of the phenytoin-loaded E15 ODFs disintegrated significantly faster than the E5-PT-G ODFs (*p* < 0.05). This finding was consistent with the results of previous studies reporting that the formulation with the higher concentration of HPMC had a significantly longer disintegration time than that of formulations containing less concentration of HPMC [27].

According to the mechanical properties and disintegration time results, the optimal formulations, E15-PT, E15-PT-PG and E15-PT-G, which exhibited good mechanical properties and faster disintegration time, were selected for further experiments.

### 3.5. Morphological Characteristics of 3D-Printed ODFs

**Figure 4:**
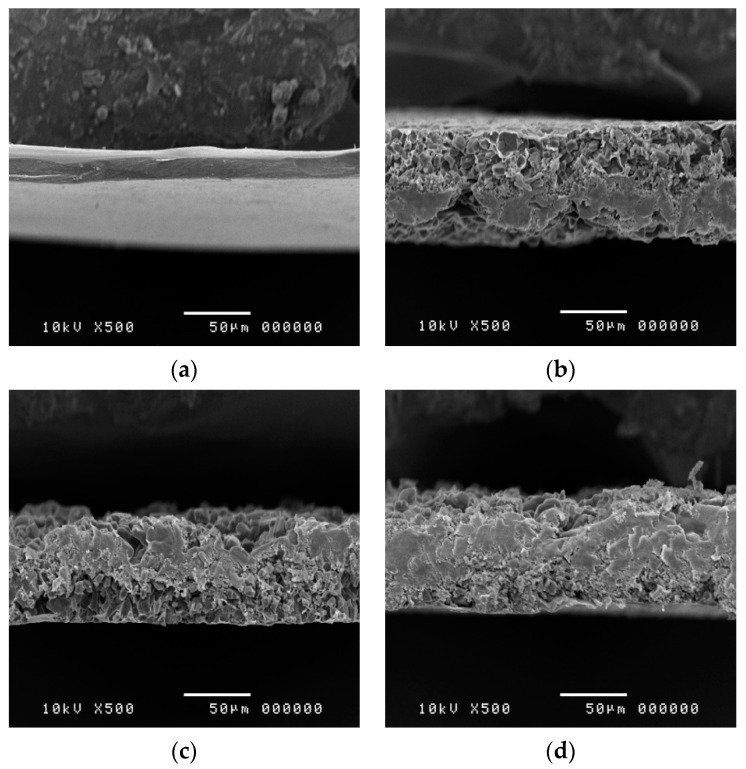
Scanning electron microscopy images of E15 (**a**), E15-PT (**b**), E15-PT-PG (**c**) and E15-PT-G (**d**).

Scanning electron microscopy images of E15, E15-PT, E15-PT-PG and E15-PT-G ODFs are shown in Figure 4. The E15-PT, E15-PT-PG and E15-PT-G ODFs demonstrated uniform and well-distributed extruded dispersions with a highly porous structure in the films. Generally, 3D printing of polymer inks with in situ evaporation of solvents has allowed fabrication of 3D porous structures with stringent requirements of rheological properties of the printing ink, e.g., high viscosity and high vapor pressure [28]. In our study, spontaneous solidification via in situ evaporation of mixed solvent of ethanol and distilled water (9:1, *v/v*) generated porosity at micro-to-nano scales. The addition of different plasticizers (PG and glycerin) did not affect the morphology of printed ODFs. In addition, there was no significant difference in the thickness observed from SEM images compared to the thickness measured by using a micrometer.

### 3.6. Phenytoin-Loading Content

In order to assess the uniformity of the printed ODFs and the robustness and precision of the film manufacturing process (3D printing process), phenytoin-loading content was evaluated. In this study, the theoretical phenytoin content in the printed ODFs size of 3.25 cm × 3.25 cm was 30 mg/film. Considering the theoretical content as 100%, the phenytoin-loading content was found to be 99.4 ± 5.2, 101.3 ± 3.8 and 99.7 ± 2.4% for E15-PT, E15-PT-PG and E15-PT-G ODFs, respectively. The results indicated that the phenytoin-loading content values of all phenytoin-loaded E15 ODFs were in agreement with the theoretical drug-loading (30 mg) and met acceptable criteria (95.0–105.0%) endorsed by the USP [18], confirming the uniformity of printed ODFs and the precision of 3D printing process of our customized syringe extrusion 3D printer.

### 3.7. In Vitro Phenytoin Release Studies

**Figure 5:**
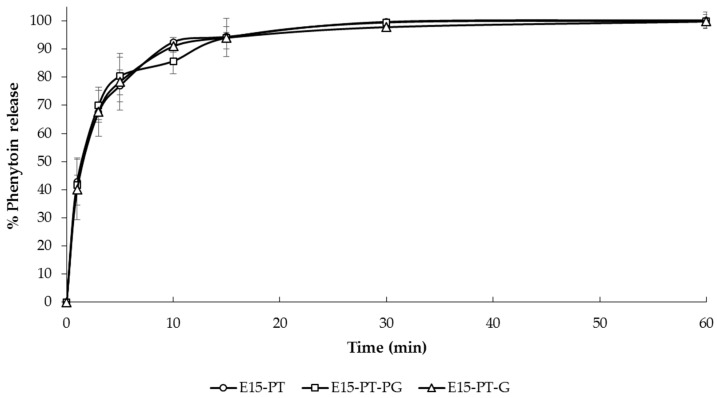
In vitro phenytoin release from E15-PT, E15-PT-G and E15-PT-PG 3D-printed ODFs (*n* = 3) in Tris with 1% *w/v* SLS buffer solution (pH 6.8).

In vitro phenytoin release experiments were carried out in Tris with 1% *w/v* SLS buffer solution pH 6.8 at 37 ± 0.5 °C. The dissolution profiles of selected 3D-printed ODFs (E15-PT, E15-PT-PG and E15-PT-G ODFs) are shown in Figure 5. All of the selected ODFs demonstrated a similar dissolution profile pattern showing the rapid phenytoin release up to 80% within 10 min, followed by a slow constant release rate to complete drug release (100% level) in 60 min. However, the dissolution rate of all selected films was not significantly different at all time points (*p* > 0.05). The rapid release behavior of phenytoin from all selected printed ODFs could be attributed to the highly porous structure of printed ODFs, fast disintegration time within 5 s, which creates more pores to allow the drug to diffuse out of the films and the hygroscopic nature of HPMC, which increases the water uptake capability of the films. According to the obtained results, this study indicated that the improvement of the dissolution rate of poorly water-soluble drugs, such as phenytoin, in the printed ODFs can be achieved. At present, there have been not many pharmaceutical products adopted 3D printing in manufacturing. In this study, we have successfully used 3D printing technology for fabricating phenytoin-loaded ODF. Although 3D printing has been used to prepare the ODFs to enhance the solubility of poorly water-soluble drugs, there has not been any research works reporting the preparation of ODFs containing phenytoin. According to the literature, Najafi et al. formulated phenytoin sodium mucoadhesive film using Carbopol 934, sodium carboxymethyl cellulose and HPMC as film formers by solvent casting method. They reported that the best formulation released 80% of the drug within 120 min [29]. However, phenytoin-loaded ODF developed by using the 3D printing technique in this study significantly enhanced drug disintegration and dissolution to less than 5 s and up to 80% in 10 min drug release, respectively, which was shown to be a novel pharmaceutical preparation possessing optimal mechanical strength and drug release profile compared with the previous reports.

## 4. Conclusions

In this study, the orodispersible films containing the required amount of phenytoin (30 mg) were successfully fabricated by 3D printing technology. The rheological properties and dimensional accuracy tests for different concentrations of HPMC E5 and HPMC E15 dispersions were performed to determine the optimal concentration and viscosity that were appropriate for the customized syringe extrusion 3D printer. The results showed that 20% *w/v* of HPMC E5 and 10% *w/v* of HPMC E15 were the most suitable polymers for incorporating drug and printing. Among all developed 3D-printed ODFs, the phenytoin-loaded E15 ODFs exhibited good physical appearance, good mechanical strength, rapid disintegration time (within 5 s) and a rapid release (up to 80% within 10 min), showing the improvement of solubility and dissolution rate of a poorly water-soluble drug, phenytoin. Overall, this study indicated that HPMC E15 is feasible to be applied as one of the 3D printing polymers for extrusion-based 3D printer and for the fabrication of orodispersible films. Additionally, this approach could be explored further for the stability of the film and the individualization of the ODFs for each patient by adjusting the volume of printing formulations.

## Figures and Tables

**Table 1:** Composition of different formulations of 3D-printed ODFs.

Formulation Code	Polymer (% *w/v*)		Phenytoin (% *w/v*)	Plasticizer (% *w/w* of Polymer)	
	HPMC E5	HPMC E15		Glycerin	Propylene Glycol
E5	20	-	15	-	-
E5-PT	20	-	15	-	-
E5-PT-PG	20	-	15	-	10
E5-PT-G	20	-	15	10	-
E15	-	10	15	-	-
E15-PT	-	10	15	-	-
E15-PT-PG	-	10	15	-	10
E15-PT-G	-	10	15	10	-

**Table 2:** Power-law model parameters and shape fidelity factor of different concentrations of HPMC E5 and HPMC E15.

Formulation	Concentration (% *w/v*)	Flow Behavior Index	Consistency Coefficient (Pa∙s*^n^*)	Shape Fidelity Factor	Diameter of Printed Filament (mm)
E5	10	0.75	2.37	1.07 ± 0.01	1.15 ± 0.17
	12.5	0.72	5.09	1.06 ± 0.02	0.97 ± 0.02
	15	0.76	7.31	1.07 ± 0.03	0.81 ± 0.02
	20	0.66	34.07	1.03 ± 0.02	0.55 ± 0.03
E15	10	0.78	18.69	1.05 ± 0.01	0.59 ± 0.07
	12.5	0.67	55.91	1.03 ± 0.01	0.56 ± 0.03
	15	NA	NA	NA	NA
	20	NA	NA	NA	NA

Note: NA (not applicable) means the printing dispersions cannot be extruded through the nozzle.

**Table 3:** Weight and thickness of 3D-printed ODFs.

Film	Weight (g ± SD)	Thickness (mm ± SD)
E5	0.0544 ± 0.0005 ^a^	0.030 ± 0.003 ^a^
E5-PT	0.0717 ± 0.0055 ^b^	0.059 ± 0.003 ^b^
E5-PT-PG	0.0730 ± 0.0032 ^b^	0.060 ± 0.003 ^b^
E5-PT-G	0.0736 ± 0.0052 ^b^	0.061 ± 0.001 ^b^
E15	0.0263 ± 0.0017 ^c^	0.013 ± 0.002 ^c^
E15-PT	0.0516 ± 0.0006 ^a^	0.056 ± 0.002 ^b^
E15-PT-PG	0.0554 ± 0.0003 ^a^	0.061 ± 0.001 ^b^
E15-PT-G	0.0549 ± 0.0002 ^a^	0.061 ± 0.001 ^b^

Note: 20% *w/v* of HPMC E5 and 10% *w/v* of HPMC E15 were used. For each test, means with the same letter are not significantly different. Thus, means with different letters, e.g., ‘a’ or ‘b’ are statistically different (*p* < 0.05).

**Table 4:** Mechanical parameters and disintegration time of 3D-printed ODFs.

Film	Puncture Strength (MPa ± SD)	Elongation (% ± SD)	Young’s Modulus (MPa ± SD)	Disintegration Time (s ± SD)
E5	2.78 ± 0.37 ^a^	6.36 ± 0.78 ^a^	216.65 ± 22.57 ^a^	ND
E5-PT	ND	ND	ND	ND
E5-PT-PG	ND	ND	ND	ND
E5-PT-G	0.32 ± 0.04 ^b^	0.44 ± 0.04 ^b^	46.97 ± 2.93 ^b^	5.51 ± 1.10 ^a^
E15	2.38 ± 0.35 ^c^	9.37 ± 1.47 ^c^	316.14 ± 25.93 ^c^	ND
E15-PT	0.38 ± 0.01 ^b^	0.64 ± 0.02 ^b^	44.16 ± 1.89 ^b^	3.39 ± 0.67 ^b^
E15-PT-PG	0.29 ± 0.04 ^b^	0.81 ± 0.16 ^b^	27.78 ± 3.55 ^b^	3.81 ± 1.33 ^b^
E15-PT-G	0.33 ± 0.02 ^b^	1.11 ± 0.34 ^b^	27.16 ± 3.54 ^b^	3.56 ± 1.20 ^b^

Note: ND (not determined) means films cannot be performed for E5-PT and E5-PT-PG ODFs, and the disintegration time of control E5 andE15 ODFs were not evaluated. For each test, means with the same letter are not significantly different. Thus, means with different letters, e.g., ‘a’ or ‘b’ are statistically different (*p* < 0.05).
